# Metal ions and nanomaterials for targeted bone cancer immunotherapy

**DOI:** 10.3389/fimmu.2025.1513834

**Published:** 2025-03-17

**Authors:** Sen Qin, YaoFeng Hu, HuaSong Luo, Wei Chu, RuCui Deng, JinLiang Ma

**Affiliations:** ^1^ Department of Orthopedics, The First Affiliated Hospital of YangTze University, Jingzhou, Hubei, China; ^2^ Department of Neurological Care Unit, The First Affiliated Hospital of YangTze University, Jingzhou, Hubei, China

**Keywords:** bone cancer, metal ions, nanomaterials, immunotherapy and tumor microenvironment, osteosarcoma

## Abstract

Bone cancer remains a significant challenge in oncology, with limited success in current therapeutic approaches, particularly immunotherapy. Emerging research highlights the potential of integrating metal ions and nanomaterials for targeted immunotherapy in bone cancer. Metal ions, including calcium, magnesium, and zinc, play a significant role in modulating immune responses within the tumor microenvironment, affecting essential pathways necessary for immune activation. Meanwhile, nanomaterials, particularly metallic nanoparticles, offer precise drug delivery and immune system modulation, improving the efficacy of immunotherapeutic agents. This review explores the synergistic effects of metal ion-nanomaterial conjugates, discussing their role in enhancing immune cell activation, particularly T-cells and macrophages, and their potential for controlled drug release. We highlight preclinical advancements in bone cancer treatment using metal ion-responsive nanoparticles, and address current challenges such as biocompatibility and toxicity. Finally, we discuss the future prospects of these technologies in personalized and precision medicine, aiming to revolutionize bone cancer immunotherapy.

## 1 Introduction

Bone cancer is a rare but aggressive form of malignancy that originates in the bone tissue, affecting both children and adults (1, 2). The two most common types of primary bone cancers are osteosarcoma and chondrosarcoma. Osteosarcoma, the most frequent, typically affects adolescents and young adults, primarily manifesting in areas of rapid bone growth, such as the distal femur, proximal tibia, and humerus (3). Osteosarcoma is highly malignant, with a propensity for early metastasis, particularly to the lungs, significantly complicating treatment (4, 5). Chondrosarcoma, in contrast, is more common in adults, typically originating in the cartilage cells and growing slowly, although certain subtypes exhibit more aggressive behavior (6, 7). The global incidence of primary bone cancers is relatively low, accounting for less than 1% of all cancers, yet the morbidity associated with these malignancies is substantial due to their aggressive nature and the likelihood of metastasis. Secondary or metastatic bone cancers, which occur when cancers from other parts of the body (like breast, prostate, or lung) spread to the bone, are more common than primary bone cancers and pose unique treatment challenges (8, 9).

Treatment of bone cancer involves a multidisciplinary approach, typically comprising surgery, chemotherapy, and radiation therapy (10–12). Bone cancer remains a significant challenge in oncology, with limited success in current therapeutic approaches, particularly immunotherapy. Traditional immunotherapy strategies, such as immune checkpoint inhibitors and monoclonal antibodies, have shown promise in various cancers. However, their application in bone cancer has been limited due to factors like poor tumor penetration, systemic toxicity, and immune resistance within the bone tumor microenvironment. Emerging research highlights the potential of integrating metal ions and nanomaterials for targeted immunotherapy in bone cancer, offering a novel approach to overcome these challenges. Metal ions, including calcium, magnesium, and zinc, play a significant role in modulating immune responses within the tumor microenvironment, and nanomaterials can enhance drug delivery and immune cell targeting. Surgical resection remains the cornerstone of treatment, especially in cases of localized osteosarcoma, where limb-sparing surgery or amputation is often required. However, despite advances in surgical techniques, recurrence rates remain high, particularly in cases of incomplete resection or microscopic residual disease. The prognosis for individuals with metastatic or recurrent disease continues to be unfavorable, despite significant improvements in survival rates among patients with localized disease due to chemotherapy, particularly with the administration of agents like doxorubicin, cisplatin, and methotrexate. Radiation therapy, although less commonly used in osteosarcoma due to its relative radioresistance, is an essential treatment modality for other types of bone cancers, such as Ewing sarcoma. One of the primary challenges in treating bone cancer is its propensity for metastasis. Osteosarcoma, in particular, has a high rate of early metastasis, with approximately 20% of patients presenting with metastases at diagnosis, primarily in the lungs (4, 13). Metastatic bone cancer is notoriously difficult to treat, and current therapeutic strategies often fall short in improving survival rates. Recurrence is another significant hurdle; even after seemingly successful treatment, bone cancer often recurs, leading to worsened prognosis (14). The limitations of conventional treatments underscore the need for innovative therapeutic approaches, such as immunotherapy, to improve outcomes for patients with bone cancer.

Immunotherapy has revolutionized the treatment of several cancers, most notably melanoma, lung cancer, and renal cell carcinoma, by harnessing the body’s immune system to recognize and destroy cancer cells. In recent years, researchers have turned their attention to the potential application of immunotherapy in the treatment of bone cancers, where traditional therapies have reached a therapeutic plateau. Tumor immunity denotes the body’s inherent capability to identify and eliminate cancerous cells. Nevertheless, cancer cells, such as those found in bone malignancies, frequently establish strategies to avoid being detected by the immune system, a phenomenon termed immune evasion. Tumor cells can manipulate the immune system through various means, such as altering the expression of immune checkpoint proteins (e.g., PD-1/PD-L1), which inhibit T-cell activity, or by creating an immunosuppressive tumor microenvironment (TME) that hinders immune cell infiltration and activation (15–17). The TME in bone cancer is particularly challenging due to the presence of bone-derived growth factors and immune-suppressive cytokines, which facilitate tumor progression and resistance to immune-based therapies (11, 18, 19).

Current immunotherapeutic strategies for bone cancer aim to counter these immune evasion tactics. One of the most promising strategies involves utilizing immune checkpoint inhibitors like PD-1 or PD-L1 inhibitors. These agents have demonstrated effectiveness in various cancer types by easing the restrictions on the immune system, thereby enabling T-cells to launch a more robust anti-tumor response. However, their efficacy in bone cancers remains limited due to the unique characteristics of the bone TME and the relatively low immunogenicity of bone cancer cells. Chimeric antigen receptor T-cell (CAR-T) therapy, which involves engineering T-cells to specifically target tumor-associated antigens, is another area of active investigation (20, 21). Although CAR-T therapy has shown impressive outcomes in specific blood malignancies, applying these achievements to solid tumors, like bone cancers, poses significant obstacles, including the necessity for efficient transport of CAR-T cells into the compact microenvironment of bone tumors (22). Despite these challenges, preclinical studies have demonstrated the potential of immunotherapy in bone cancer. For instance, dendritic cell vaccines and adoptive T-cell transfer therapies are being explored as ways to enhance immune recognition of bone cancer cells. Moreover, therapies involving cytokines that enhance the activation and multiplication of immune cells, including interleukin-2 (IL-2) and interleukin-12 (IL-12), are currently under investigation for their potential to trigger immune responses in bone cancer. While immunotherapy is still in its early stages in bone cancer, these approaches offer promising avenues for improving outcomes, particularly when combined with emerging technologies such as metal ions and nanomaterials.

Nanotechnology has emerged as a transformative field in cancer treatment, offering new possibilities for drug delivery, imaging, and immunomodulation (23, 24). Nanomaterials, due to their small size and large surface area-to-volume ratio, possess unique physicochemical properties that can be exploited for therapeutic purposes. In cancer therapy, nanoparticles can be functionalized to target specific tumor cells, deliver therapeutic agents more efficiently, and reduce systemic toxicity compared to conventional treatments. In the realm of immunotherapy for bone cancer, there has been a notable interest in metal-based nanomaterials, such as gold, silver, and iron oxide nanoparticles, because of their potential to boost immune responses, optimize drug delivery, and modify the tumor microenvironment (25, 26). These nanostructures can be specifically designed to transport immune-stimulating drugs, antigens, or metal ions directly to the location of the tumor, which helps reduce off-target effects while maximizing the effectiveness of treatments. A case in point is gold nanoparticles, which have been extensively researched for their role as carriers for both anticancer medications and immune checkpoint inhibitors. With their excellent biocompatibility and customizable characteristics, gold nanoparticles can be modified with particular ligands to enhance their absorption by both tumor and immune cells (27, 28).

Metal ions like calcium, magnesium, and zinc are essential for cellular signaling and the regulation of the immune system (29–31). These ions can directly impact the tumor microenvironment, affecting the growth of cancer cells and the functionality of immune cells. For example, zinc ions play a role in signaling within immune cells and have demonstrated an influence on the activities of important immune cell types, including T-cells and macrophages (32–34). Magnesium ions, on the other hand, are critical for maintaining the integrity of the immune system and have been implicated in T-cell activation and proliferation (35, 36). In bone cancer, metal ions can modulate the immune response, either promoting or inhibiting tumor progression depending on their concentration and interaction with the tumor microenvironment. The combination of metal ions and nanomaterials offers a synergistic approach to targeted bone cancer immunotherapy. By integrating metal ions into nanomaterial-based systems, researchers can develop innovative therapies that not only target cancer cells but also modulate immune responses. Metal ion-doped nanoparticles, for example, can be designed to release specific metal ions in response to the acidic tumor microenvironment, thus enhancing the immune system’s ability to recognize and attack cancer cells. Furthermore, these systems have the potential to transport immunostimulatory agents or immune checkpoint inhibitors straight to the tumor location, which enhances the body’s inherent immune reaction to bone cancer.

Metal ions and nanomaterials show great potential in the field of bone cancer immunotherapy. By utilizing the unique properties of these materials, researchers can develop more effective and targeted therapies to tackle challenges such as metastasis, recurrence, and immune evasion in bone cancer. As research advances, the integration of nanotechnology with immunotherapy brings new hope for combating this aggressive disease.

## 2 Metal ions in cancer immunotherapy

### 2.1 Biological functions of metal ions in tumor microenvironment

Metal ions play pivotal roles in maintaining cellular function, and their involvement in signaling pathways is essential for numerous physiological processes, including immune regulation. In the TME, the levels and availability of metal ions can significantly affect tumor progression and immune responses. Crucial metal ions, including calcium, magnesium, and zinc, play a vital role in the regulation of various processes such as cell division, gene expression, and apoptosis. In the context of cancer, these metal ions influence both tumor cells and immune cells, thereby impacting tumor growth and the efficacy of immunotherapies.

Calcium ions (Ca^2+^) are vital for cellular signaling, including the activation of immune cells like T-cells. Upon activation, T-cells experience a rapid influx of calcium ions, which facilitates signal transduction necessary for cytokine production and cell proliferation (37, 38). Calcium signaling also modulates the activity of key transcription factors such as NFAT (Nuclear Factor of Activated T-cells), which is crucial for T-cell activation and immune responses. However, in cancer, calcium dysregulation can promote tumor survival and metastasis. Elevated intracellular calcium levels in cancer cells are linked to increased proliferation, angiogenesis, and resistance to apoptosis, all of which contribute to cancer progression (39, 40).

Magnesium ions (Mg^2+^) are another essential component of cellular function, playing a crucial role in DNA repair, ATP metabolism, and protein synthesis (35, 41). Magnesium is indispensable for maintaining normal immune function, particularly for T-cell activity. Magnesium deficiency has been associated with impaired immune responses, while adequate magnesium levels are critical for T-cell activation and proliferation (36). In cancer, magnesium homeostasis is often disrupted, leading to either insufficient or excessive magnesium levels in the TME. Such imbalances can contribute to cancer cell survival and immune evasion by creating an environment that suppresses anti-tumor immunity.

Zinc ions (Zn^2+^) are essential for the functioning of more than 300 enzymes and numerous transcription factors. Zinc plays a particularly important role in the immune system by modulating cytokine production, T-cell maturation, and macrophage activity. A lack of zinc hampers the effectiveness of both innate and adaptive immune systems, resulting in reduced anti-tumor immunity. Conversely, high levels of zinc in the TME can enhance the production of pro-inflammatory cytokines, thereby promoting an immune-suppressive microenvironment that favors tumor growth.

Dysregulation of metal ions within the TME profoundly impacts cancer progression. Imbalances can enhance tumor cell proliferation, resistance to apoptosis, angiogenesis, and metastasis. For instance, altered zinc homeostasis in various cancers supports tumor growth through sequestration by cancer cells, while aberrant calcium signaling promotes invasive behavior and resistance to apoptosis in tumor cells. Moreover, improper regulation of metal ions may impair immune cell function, weakening anti-tumor immunity and fostering an environment conducive to cancer development.

### 2.2 Metal ions for immune modulation in bone cancer

Given the critical role of metal ions in immune function, researchers have been exploring ways to harness metal ions for immune modulation in cancer therapy, particularly in bone cancers like osteosarcoma. Metal ions can directly influence immune cell activity, making them promising candidates for enhancing the efficacy of cancer immunotherapies. The activation and functioning of T-cells, which are key components of adaptive immunity, rely heavily on magnesium ions. Magnesium acts as a cofactor for numerous enzymes involved in ATP production and DNA replication, both of which are essential for T-cell proliferation. Additionally, magnesium helps stabilize the structure of major histocompatibility complex (MHC) molecules, which are responsible for presenting tumor antigens to T-cells (42, 43). This makes magnesium essential for effective T-cell responses against tumors. Studies have shown that restoring magnesium levels in magnesium-deficient cancer patients can improve T-cell activity and enhance the efficacy of immune checkpoint inhibitors like PD-1/PD-L1 inhibitors. Zinc ions are critical regulators of cytokine production, particularly in the activation of macrophages and T-helper cells. Zinc modulates the activity of transcription factors such as NF-κB (nuclear factor kappa-light-chain-enhancer of activated B cells), which controls the expression of pro-inflammatory cytokines like TNF-α and IL-6 (44, 45). These cytokines are crucial in fostering anti-tumor immune responses by activating and recruiting immune cells within the TME. Nevertheless, a deficiency in zinc hampers the production of these cytokines, which diminishes immune surveillance and permits tumors to evade detection by the immune system. Research has indicated that zinc supplementation within the TME can restore cytokine production and improve the anti-tumor immune response in numerous preclinical cancer models. Calcium ions are vital for the trafficking of immune cells, especially the migration of T-cells to tumor locations. The signaling of calcium modulates the expression of adhesion molecules and chemokine receptors on immune cell surfaces, facilitating their movement towards the tumor (46, 47). In instances of bone cancer, where the TME is frequently immunosuppressive, restoring typical calcium signaling may enhance immune cell infiltration into the tumor, thus boosting the effectiveness of immunotherapies.

### 2.3 Metal ion transporters and immune pathways

The biological effects of metal ions in the TME are mediated by specialized metal ion transporters, which regulate the intracellular and extracellular concentrations of these ions. In cancer cells, these transporters are often dysregulated, leading to altered metal ion homeostasis that promotes tumor survival and immune evasion. Magnesium homeostasis is regulated by TRPM7 (transient receptor potential cation channel subfamily M member 7), a magnesium ion channel that plays a role in controlling intracellular magnesium levels (48, 49). TRPM7 is often overexpressed in cancer cells, including bone cancers, where it promotes cell survival, proliferation, and metastasis. In the context of immunotherapy, targeting TRPM7 could restore normal magnesium levels in the TME, thereby enhancing immune cell activity and improving the efficacy of T-cell-based therapies. Zinc is transported into and out of cells by ZIP (Zrt/Irt-like Protein) and ZnT (Zinc Transporter) families of proteins. Dysregulation of these transporters in cancer cells leads to altered zinc homeostasis, which can affect both tumor cell survival and immune cell function. In bone cancer, overexpression of ZIP4 has been linked to enhanced tumor growth and metastasis. Modulating zinc transporter activity could restore normal zinc levels in the TME, leading to improved immune responses against bone tumors.

Calcium ions are tightly regulated by a network of transporters, including PMCA (Plasma Membrane Calcium ATPase) and NCX (Na_+_/Ca^2+^ exchangers), which control intracellular calcium concentrations (50, 51). Dysregulation of these transporters in cancer cells leads to altered calcium signaling, promoting tumor survival and immune evasion. In bone cancer, targeting calcium transporters may restore normal calcium signaling in immune cells, leading to enhanced infiltration and activation of T-cells in the TME. The immune-related pathways affected by metal ion dysregulation include key signaling cascades such as NF-κB and MAPK. NF-κB plays a crucial role in regulating immune responses by managing the expression of cytokines, chemokines, and adhesion molecules that are essential for the trafficking and activation of immune cells. In cancer cells, disrupted signaling of calcium, magnesium, and zinc can trigger NF-κB activation, which results in the secretion of immunosuppressive cytokines that aid in tumor survival. Additionally, the MAPK (mitogen-activated protein kinase) pathway, which governs cell proliferation and survival, is also affected by levels of metal ions. Adjusting the function of metal ion transporters may help restore the normal signaling of NF-κB and MAPK in immune cells, thereby boosting their capability to combat tumors.

## 3 Nanomaterials in targeted cancer therapy

### 3.1 Types of nanomaterials used in cancer therapy

**Figure 1:**
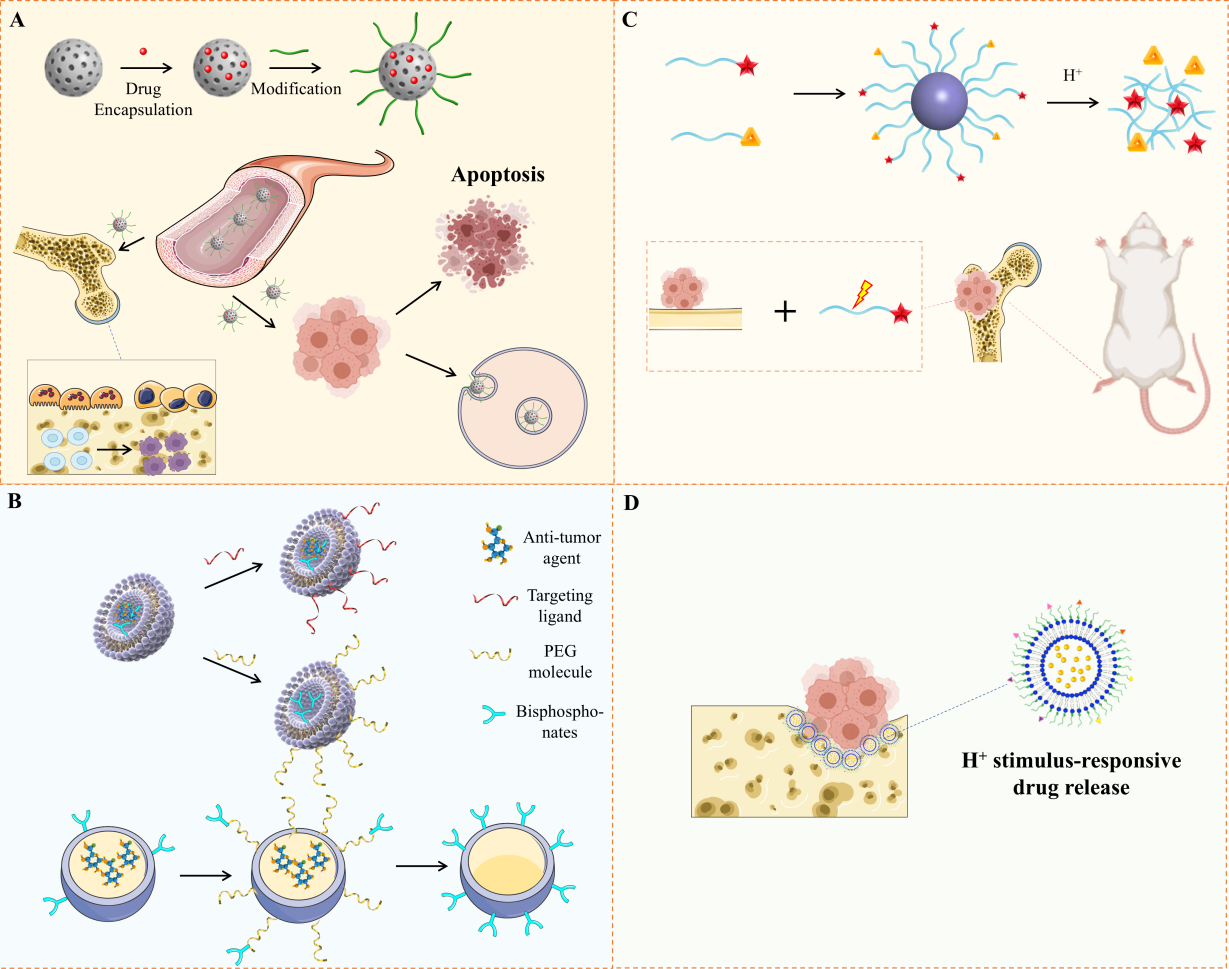
Illustration of Nanoplatforms for Bone Metastasis Based on Drug Delivery. **(A)**The diagram illustrates a nanoplatform engineered to target both bone and tumors, improving drug concentration at specific sites, inducing tumor cell death, and inhibiting osteoclast function. **(B)** It presents various types of nanocarriers developed for precise drug delivery to bone tumors. **(C)** Alendronate-modified micelles, formed from two polymers, incorporating alendronate, catechol, and bortezomib. **(D)** Alendronate-coated Ag_2_S nanoparticles, loaded with doxorubicin, were created for bone tumor therapy and have shown the ability to bind hydroxyapatite.

Nanomaterials have gained significant attention in the realm of cancer therapy, primarily due to their distinctive physicochemical attributes. Their diminutive size, customizable surface chemistry, and capacity to engage with biological systems at the molecular level render them remarkably effective for cancer diagnosis and treatment. Notably, extensive research has been conducted on the role of nanomaterials in targeted drug delivery, with the aim of reducing off-target toxicity while maximizing therapeutic effectiveness (**Figure 1**). In this discussion, we categorize the various nanomaterials utilized in cancer therapy, highlighting their specific properties and benefits. Metallic nanoparticles, especially those composed of gold, silver, iron oxide, and platinum, are the focus of much research in cancer treatment, attributed to their excellent biocompatibility, functionalization ease, and capability to enhance drug delivery (52, 53). Gold nanoparticles (AuNPs), for instance, are highly stable and have a high surface area that allows for conjugation with drugs, antibodies, or other molecules for targeted delivery. They also exhibit photothermal properties, meaning they can convert light energy into heat, a feature that can be harnessed for photothermal therapy (PTT) to selectively destroy cancer cells. Silver nanoparticles (AgNPs) possess well-documented antimicrobial characteristics and have additionally demonstrated potential in cancer treatment, primarily because of their capacity to produce reactive oxygen species (ROS) that can trigger apoptosis in cancerous cells. Similarly, iron oxide nanoparticles, frequently employed in magnetic resonance imaging (MRI), have been engineered to function as magnetic drug carriers, enabling the targeted delivery of therapeutics to tumors through the guidance of magnetic fields. Lastly, platinum nanoparticles are investigated for their role in chemotherapy, especially as carriers of platinum-based drugs like cisplatin, enhancing their solubility and reducing systemic toxicity.

Nanocomposites are hybrid materials that combine different types of nanomaterials to create multifunctional systems. These can be made from combinations of metallic nanoparticles with polymers, liposomes, or hydrogels to improve their pharmacokinetics and targeting abilities. For example, gold-liposome composites can encapsulate chemotherapy drugs inside liposomes while using the gold component for photothermal therapy (54, 55). Such systems facilitate the concurrent administration of various therapeutic agents, such as drugs, genes, or immune modulators, thereby improving treatment efficacy. Additionally, nanocomposites can provide controlled release of drugs, prompted by stimuli like pH variations, temperature fluctuations, or magnetic fields, which frequently characterize the tumor microenvironment. Among the most recognized carbon-based nanomaterials utilized in cancer therapy are carbon nanotubes (CNTs) and graphene oxide (GO) (56). CNTs are cylindrical nanostructures known for their remarkable mechanical, electrical, and thermal properties. Their large surface area and capability to infiltrate cellular membranes make CNTs outstanding candidates for applications in drug delivery, gene therapy, and photothermal therapy. Graphene oxide, with its two-dimensional structure, provides an extensive surface for drug loading and has been shown to improve the solubility of hydrophobic drugs. Graphene’s unique properties allow it to interact with biological membranes and deliver therapeutic agents directly into the cells, making it a valuable tool in cancer treatment, including in bone cancer applications (57).

Dendrimers are a type of highly branched, tree-shaped polymers that can be created with exact control regarding their dimensions, form, and surface properties. The organized nature of these structures offers numerous locations for drug loading, and their surfaces can be modified to selectively target specific receptors present on cancer cells (58). Dendrimers have shown great promise in delivering small molecules, siRNA, and even proteins to cancer cells, and their ability to traverse biological barriers makes them particularly useful for targeted delivery in solid tumors, including bone cancers. The unique properties of nanomaterials make them suitable for cancer therapy. Their small size (typically 1-100 nm) allows them to circulate through the bloodstream and penetrate tumor tissues more effectively than larger molecules. This is particularly important in bone cancer, where the dense extracellular matrix can limit the penetration of conventional drugs. Moreover, different ligands, including antibodies, peptides, or aptamers, can be utilized to functionalize the surfaces of nanomaterials, allowing for precise targeting of particular receptors on cancer cells, thereby enhancing active targeting. Their ability to carry multiple therapeutic agents or contrast agents also enables combination therapies and theranostics, where treatment and imaging can be combined in a single system.

### 3.2 Nanomaterial-mediated targeting of bone cancer cells

Extensive research has been conducted on nanomaterials due to their potential to selectively target cancerous cells, including those found in bone tissue. In cases of bone malignancies such as osteosarcoma and Ewing sarcoma, standard chemotherapy frequently results in considerable side effects and restricted effectiveness, primarily attributed to inadequate drug distribution and the challenge of reaching metastatic or resistant cancer cells. Nanomaterials present a promising solution by facilitating targeted drug delivery, which allows therapeutic agents to be administered directly to cancer cells while reducing harm to healthy tissues and addressing mechanisms of drug resistance. Passive targeting exploits the unique characteristics of the tumor microenvironment, particularly the enhanced permeability and retention (EPR) effect (59, 60). Tumors, including bone cancers, typically exhibit leaky vasculature with large gaps between endothelial cells, allowing nanoparticles to accumulate within the tumor tissue. Additionally, tumors have poor lymphatic drainage, which means that once nanomaterials enter the tumor, they are retained for longer periods. This passive targeting mechanism allows nanoparticles to selectively accumulate in tumor tissues, increasing the local concentration of the drug while reducing systemic exposure. For instance, liposomal formulations of chemotherapy drugs like doxorubicin have shown improved efficacy in osteosarcoma by enhancing drug accumulation in the tumor while reducing cardiotoxicity (61, 62).

Active targeting involves functionalizing the surface of nanomaterials with ligands that can specifically bind to receptors overexpressed on the surface of cancer cells. In bone cancer, potential targets include bone morphogenetic proteins (BMPs), integrins, and vascular endothelial growth factor (VEGF) receptors, all of which are overexpressed in osteosarcoma and other bone tumors. Nanoparticles that are functionalized with RGD peptides are capable of selectively binding to αvβ3 integrin receptors, which are prominently expressed in osteosarcoma cells, thus guaranteeing the direct delivery of the therapeutic agents to these cancer cells (63). Likewise, nanoparticles modified with antibodies can aim at specific antigens present on cancer cells, thereby improving the therapy’s selectivity. Beyond their role in drug delivery, nanomaterials can also transport immune modulators to the tumor site, boosting the body’s inherent immune response against bone cancer cells. For instance, gold nanoparticles can be functionalized with immunostimulatory molecules such as CpG oligonucleotides or anti-PD-L1 antibodies, which can activate dendritic cells or block immune checkpoints, respectively. By delivering these immune modulators directly to the tumor, nanomaterials can enhance the effectiveness of immunotherapy in bone cancer. Furthermore, nanomaterials can be engineered to release their therapeutic payload in response to specific stimuli in the tumor microenvironment, such as low pH or high levels of reactive oxygen species, ensuring that the drug is released only in the vicinity of the cancer cells, further minimizing off-target effects.

The unique structure of bone tissue poses significant challenges for drug delivery, especially in metastatic or recurrent bone cancers. The dense extracellular matrix and the hypoxic, immunosuppressive tumor microenvironment hinder the efficacy of standard treatments. Nanomaterials offer multiple advantages: Their small size and large surface area allow nanoparticles to penetrate dense bone matrix more efficiently and can be engineered to target areas with high osteoclast activity, enhancing accumulation in bone lesions. The functionalization of nanomaterials with ligands that bind to bone-specific receptors increases therapeutic agent retention in bone tissue, reducing dosing frequency and side effects. For example, bisphosphonate-functionalized nanoparticles are designed to target osteoclasts, ensuring preferential delivery to bone metastases. Moreover, nanomaterials enable the simultaneous delivery of diverse therapeutic agents—chemotherapy drugs, immune modulators, and osteogenic factors—which promote bone regeneration while treating cancer. This is crucial for maintaining bone integrity and preventing complications like fractures in bone cancer cases.

## 4 Synergistic role of metal ions and nanomaterials in bone cancer immunotherapy

**Figure 2:**
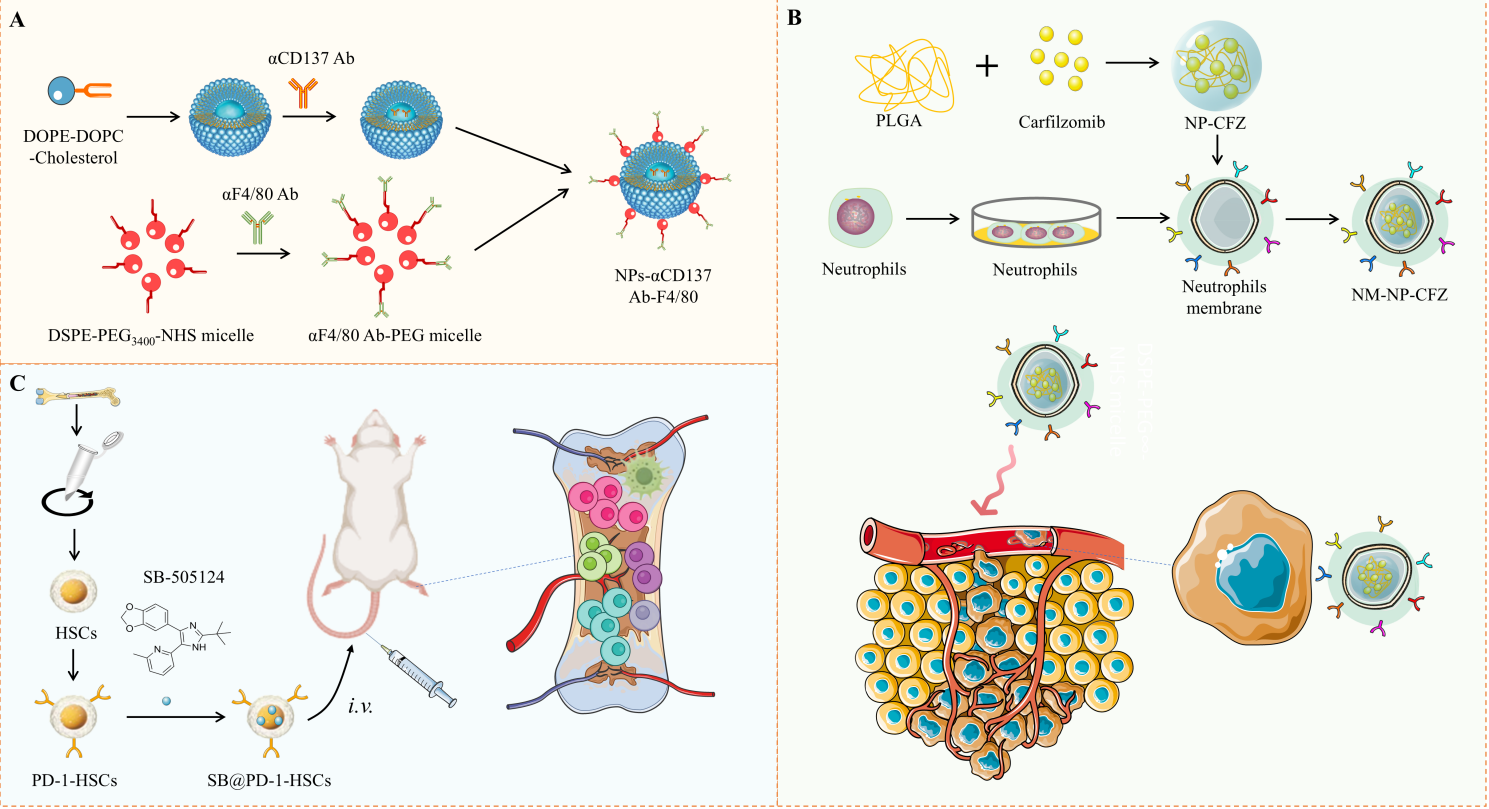
Schematic Illustration of Nanoplatforms for Immunomodulation of Bone Metastasis. **(A)** A schematic shows the construction of F4/80-targeted liposomal nanoparticles loaded with anti-CD137 antibodies. **(B)** Neutrophil-mimicking nanoparticles carrying the second-generation proteasome inhibitor carfilzomib, designed to target circulating tumor cells and distant metastases. **(C)** Depicts the mechanism of PD-1-engineered hematopoietic stem cells, which bind to PD-L1+ cells at metastatic sites to inhibit bone metastasis.

The integration of metal ions and nanomaterials in the realm of cancer treatment is a rapidly evolving area, particularly regarding difficult-to-treat cancers like bone tumors. The effectiveness of immunotherapy for bone cancer has been somewhat constrained due to the immune-suppressing characteristics of the TME and the difficulties associated with the direct delivery of immunotherapeutic agents to the tumor location. Metal ions have the potential to influence immune responses, while nanomaterials are capable of allowing for precise delivery, presenting a promising avenue for the creation of new synergistic treatment approaches. When combined, metal ion-nanomaterial conjugates can play a dual role: enhancing immune activation while delivering therapeutic agents to the tumor site with precision (**Figure 2**). In this section, we will explore the synergistic effects of these materials in bone cancer immunotherapy, focusing on immune activation, controlled drug delivery, and emerging photothermal and photodynamic therapies.

### 4.1 Metal ion-nanomaterial conjugates for immune activation

The use of metal ion-nanomaterial conjugates in immunotherapy is a promising strategy for enhancing immune activation against cancer cells. Ions of metals like calcium, magnesium, zinc, and copper play well-established roles in regulating the function of immune cells. These ions can directly influence the activity of immune cells, including T-cells, macrophages, and dendritic cells, all of which are crucial for mounting an effective anti-tumor immune response. However, delivering these ions to the tumor microenvironment in a controlled manner is a challenge that can be overcome by conjugating them to nanomaterials. Nanomaterials, such as metallic nanoparticles, liposomes, and polymeric nanoparticles, provide an ideal platform for the controlled release of metal ions. By conjugating metal ions to these nanocarriers, researchers can ensure that the ions are delivered directly to the tumor site, where they can modulate the immune microenvironment and enhance the efficacy of immunotherapies. These metal ion-nanomaterial conjugates can be engineered to release their payload in response to specific stimuli in the TME, such as pH changes, hypoxia, or the presence of specific enzymes, ensuring that immune activation occurs precisely where it is needed.

One of the most exciting applications of metal ion-nanomaterial conjugates is in the delivery of immune checkpoint inhibitors or tumor antigens. Immune checkpoint inhibitors, such as anti-PD-1 or anti-CTLA-4 antibodies, have revolutionized cancer immunotherapy by blocking inhibitory signals that prevent T-cells from attacking cancer cells. However, their systemic administration can lead to immune-related adverse events. To mitigate these effects, researchers have developed hybrid nanocarriers that conjugate immune checkpoint inhibitors with metal ions, enhancing the localized immune activation at the tumor site while minimizing off-target effects. For example, gold nanoparticles (AuNPs) conjugated with copper ions and loaded with anti-PD-L1 antibodies have been shown to effectively target and modulate the immune microenvironment in bone cancer models (64, 65). The gold-copper ion conjugates not only delivered the immune checkpoint inhibitors but also modulated T-cell activation by influencing intracellular calcium signaling, a crucial step in T-cell proliferation and function. This dual action resulted in enhanced tumor infiltration by cytotoxic T-cells and improved anti-tumor responses. Additionally, metal ion-nanomaterial conjugates can deliver tumor antigens to dendritic cells (DCs), key players in antigen presentation and the initiation of immune responses. For example, zinc-doped mesoporous silica nanoparticles have been developed to deliver tumor-associated antigens along with zinc ions, which modulate DC maturation and cytokine secretion. In bone cancer, such systems could significantly enhance the presentation of tumor antigens to T-cells, amplifying the anti-tumor immune response.

### 4.2 Metal ion-responsive nanoparticles in immunotherapy

One of the critical advantages of metal ion-based nanomaterials is their ability to be engineered as metal ion-responsive nanoparticles. These nanoparticles are designed to release their therapeutic payload in response to specific signals in the tumor microenvironment, such as changes in pH, oxidation-reduction potential, or enzyme activity. In the acidic surroundings typical of various tumors, such as bone cancers, these systems responsive to metal ions can be activated to discharge immune modulators, chemotherapy drugs, or metal ions that boost immune reactions. The acidic TME of bone cancer is an ideal target for pH-responsive nanoparticles. For example, calcium phosphate nanoparticles have been developed to degrade and release calcium ions in response to the acidic conditions found in tumors (66–68). Calcium ions play a pivotal role in T-cell activation, and their controlled release in the TME can enhance T-cell responses against cancer cells. Research on osteosarcoma demonstrated that these nanoparticles composed of calcium phosphate not only boost T-cell infiltration within the tumor environment but also increase the effectiveness of immune checkpoint inhibitors when administered together. By controlling the release of calcium ions exclusively in the acidic tumor microenvironment, the nanoparticles reduce unintended effects and enhance the precision of the immune response.

Similarly, the translational potential of combining nanotechnology with immunotherapy is also significant. Multidisciplinary strategies that integrate nanotechnology with immunotherapy are proving to be pivotal in overcoming the limitations of current treatments for both bone and soft tissue cancers. A recent study by Mercatali et al. provides a critical overview of advancements in cancer nanotechnology, specifically focusing on drug delivery systems (69). These systems are designed to enhance the pharmacokinetics and targeting of therapeutic agents, ensuring better drug distribution within tumors while reducing systemic toxicity. This work aligns with the potential of combining nanotechnology and immunotherapy in improving the efficacy of bone cancer treatments, as it demonstrates how nanomaterials can not only enhance drug delivery but also modulate immune responses, creating a synergistic effect for targeted cancer therapy”.

In addition to pH-responsive systems, redox-responsive nanoparticles that release metal ions in response to the high levels of reactive oxygen species (ROS) present in many tumors have been developed (70). Iron oxide nanoparticles, for example, can release iron ions in response to oxidative stress, enhancing the production of ROS within cancer cells. This ROS generation not only induces cancer cell death but also activates immune cells in the TME. In a bone cancer model, iron oxide nanoparticles were used to deliver anti-PD-1 antibodies alongside iron ions, resulting in enhanced T-cell activation and tumor regression. Research has also investigated magnesium-doped nanomaterials for their involvement in activating T-cells. Magnesium plays a crucial role in T-cell receptor (TCR) signaling and the proliferation of T-cells. Magnesium-doped silica nanoparticles can release magnesium ions in response to TME-specific triggers, boosting T-cell function and improving the overall immune response. These nanoparticles have been studied in combination with adoptive T-cell therapies, where T-cells engineered to target bone cancer cells are infused into the patient. The magnesium release enhanced the proliferation and cytotoxicity of the infused T-cells, leading to improved outcomes in preclinical models of bone cancer.

### 4.3 Photothermal and photodynamic effects of metal nanoparticles

Metal nanoparticles have unique properties that can enhance cancer treatment through photothermal therapy and photodynamic therapy. Both methods use these nanoparticles to kill tumor cells by producing heat or reactive oxygen species (ROS). Combining these techniques with immunotherapy can destroy tumor cells and enhance long-term anti-tumor immune responses.

PTT utilizes nanoparticles, such as gold nanoparticles (AuNPs), to absorb near-infrared (NIR) light and convert it into heat. This local heat-induced hyperthermia selectively targets cancer cells while sparing healthy tissue. This targeted approach is particularly useful for bone cancer, where traditional treatments, such as radiotherapy, can damage healthy bones. PTT combined with immunotherapy improves treatment outcomes. The heat generated by gold nanoparticles (AuNPs) can stimulate dying cells to release tumor antigens. These antigens are subsequently captured by dendritic cells, which present them to T cells, thereby boosting the immune response against tumors (71, 72). Research indicates that the combination of gold nanoparticles with immune checkpoint inhibitors enhances tumor destruction and activates the immune system, resulting in improved tumor regression and survival, in comparison to each treatment used independently (73, 74).

PDT uses photosensitizers, which produce ROS when exposed to light. Metal nanoparticles such as zinc oxide and titanium dioxide are effective photosensitizers because of their ability to generate ROS. In bone cancer, PDT induces oxidative stress, leading to tumor cell death and the release of dangerously relevant molecular patterns (DAMPs) that further activate the immune system. Studies of zinc oxide nanoparticles have shown that PDT can destroy tumors and stimulate natural killer cells (NK) and T cells. When PDT is used in combination with immune checkpoint blockade, it can enhance immune activation, leading to a stronger anti-tumor response and prolonged protection against recurrence.

Both PTT and PDT cause immunogenic cell death, release tumor antigens and inflammatory signals, and activate the immune system. When used in conjunction with immunomodulators such as checkpoint inhibitors, these therapies can transform the tumor microenvironment (TME) from immunosuppression to immune activation, significantly improving the effectiveness of immunotherapy for bone cancer.

## 5 Mechanisms of action: how metal ions and nanomaterials interact with immune cells

In bone cancer immunotherapy, understanding the intricate interactions between metal ions, nanomaterials, and immune cells is key to advancing effective therapeutic strategies. TME in bone cancers such as osteosarcoma and chondrosarcoma are often immune-suppressive, preventing effective anti-tumor immune responses. Both macrophages and DCs play central roles in initiating and regulating immune responses, while T-cells serve as primary effectors of adaptive immunity. Metal ions such as calcium, magnesium, and zinc are critical regulators of immune cell function, and when combined with nanomaterials, they can enhance immune activation, improve antigen presentation, and promote more effective anti-tumor immunity. In this section, we will explore how metal ions and nanomaterials interact with these key immune cells and discuss their impact on enhancing the body’s natural defenses against bone cancer (**Table 1**) (75–86).

**Table 1:** Ions-based Nanomaterials for immunotherapy.

Ions based nanomaterials	Immunogens	Function	Immune effects	Ref
MnP-PEG nanoclusters		stimulate the cGAS-STING pathway	Activated` the immature bone marrow-derived dendritic cells and promoted cytotoxicity of T cells in the tumor	(74)
Aluminum (oxy) hydroxide nano-sticks	Ovalbumin	Adjuvant	Released more IL-1β and induced significantly higher levels of serum anti-OVA IgG and IgG1 than OVA-adsorbed Al-hydrogel	(75)
Poly caprolactone-PEG-Al nanoparticle	Tetanus toxoid	Adjuvant	Induced higher levels of IFN-γ and IL-4 and produced strong antibody response	(76)
Aluminum hydroxide	Ovalbumin	Carrier	Increased significantly the expression of surface molecules CD80 and CD86, promoted the activation of tumor specific T cells	(77)
OVA@Mn-DAP nanoparticles	Ovalbumin	Imaging agent	Inhibited growth of already-established tumors	(78)
Gold nanoparticle	Endogenous EDB self-antigens	Endogenous EDB self-antigens	Cross-presentation of the antigen in professional DCs was mediated effectively	(79)
Mesoporous ZnO nano-capsule	Ovalbumin	Adjuvant/Carrier	Enhanced expression of antigen specific T-cells and increased IgG2a or IgG2b levels	(80)
Lipid-coated zinc phosphate hybrid	Multi-peptide (TRP2180-188 and HGP10025-33)	Adjuvant/Carrier	Increased CD8+ T cell response	(81)

### 5.1 Interaction with macrophages and dendritic cells

Dendritic cells and macrophages are crucial in orchestrating immune responses, particularly within the TME. Macrophages are highly plastic immune cells that can be polarized into different functional states depending on environmental signals. In cancer, macrophages can promote or inhibit tumor growth, depending on their polarization state. Tumor-associated macrophages (TAMs) typically exhibit an M2 phenotype, often associated with immunosuppression and tumor progression. However, metastasis of macrophages towards a pro-inflammatory and anti-tumor M1 phenotype can significantly enhance the immune response against cancer (87). Dendritic cells are specialized antigen-presenting cells that are responsible for capturing, processing, and presenting tumor antigens to T cells, thereby initiating adaptive immune responses (88). Both macrophages and DCs are essential for activating T cells and promoting robust anti-tumor immunity.

Certain metal ions, including zinc and copper, have the ability to regulate the polarization of macrophages. It is well-established that zinc ions affect NF-κB signaling pathways, leading to an increase in pro-inflammatory cytokine production, such as IL-6 and TNF-α, thereby steering macrophage polarization towards the M1 phenotype. This switch is critical in converting TAMs from an immune-suppressive to an immune-activating state. Zinc-doped nanoparticles, for example, have been shown to polarize macrophages towards the M1 phenotype in the TME, enhancing phagocytic activity and cytokine production, which leads to a more robust immune attack on cancer cells (89). Similarly, copper ions have been shown to enhance ROS production in macrophages, further promoting the M1 phenotype. These metal ions can be delivered in a controlled manner using metal-doped nanoparticles that release their ion payloads specifically within the TME, ensuring that macrophages in proximity to the tumor are activated. This approach can be particularly effective in the bone TME, where TAMs are abundant and play a key role in suppressing immune responses. By reprogramming these TAMs into pro-inflammatory M1 macrophages, metal ion-based therapies can stimulate a more effective anti-tumor immune response.

Dendritic cells (DCs) are critical for bridging the innate and adaptive immune systems, as they capture antigens and present them to T-cells, leading to T-cell activation. Metal-based nanoparticles, such as gold nanoparticles or zinc oxide nanoparticles, can enhance DC activation by improving antigen uptake, processing, and presentation. For example, gold nanoparticles functionalized with tumor antigens can be taken up by DCs, leading to enhanced MHC class I and MHC class II presentation to CD8+ and CD4+ T-cells, respectively. This process boosts the immune system’s ability to recognize and attack tumor cells. Additionally, metal ions can directly influence DC function. Calcium ions, for example, are known to regulate several key processes in DC activation, including cytokine production and antigen presentation. Calcium ion-releasing nanoparticles, such as calcium phosphate nanocarriers, can be used to modulate intracellular calcium levels in DCs, enhancing their maturation and improving their capacity to present antigens to T-cells. This is particularly important in bone cancer, where efficient antigen presentation is critical for eliciting a strong anti-tumor immune response.

Metal ion-based nanoparticles promote dendritic cell (DC) migration toward lymph nodes, enhancing interactions with naive T-cells and the activation of adaptive immune responses. For instance, zinc-ion-loaded nanoparticles have been shown to increase CCL21 production, a chemokine that attracts DCs to lymphoid tissues. By improving DC migration and antigen presentation, these nanomaterials enhance the overall immune response against bone tumors. Macrophages and DCs are also involved in cytokine production, crucial for establishing a pro-inflammatory TME conducive to tumor destruction. Metal ions such as zinc and calcium boost cytokine production from these cells, particularly pro-inflammatory cytokines like IFN-γ, TNF-α, and IL-12, which activate natural killer (NK) cells and cytotoxic T-cells. Zinc oxide nanoparticles, for example, have been found to increase IL-12 production, enhancing T-cell and NK cell activity. By modulating the cytokine milieu in the TME, metal ions and nanomaterials shift the balance from immune suppression to activation, improving the body’s ability to combat bone cancer.

### 5.2 Impact on T-cell activation and proliferation

T-cells are the primary effectors of adaptive immunity and are critical for the success of immunotherapy in cancer treatment. In bone cancer, T-cells face several challenges, including exhaustion, immune suppression, and poor infiltration into the TME. Metal ions and nanomaterials can address these challenges by enhancing T-cell activation, promoting proliferation, and preventing T-cell exhaustion.

Metal ions such as calcium, magnesium (Mg^2+^), and zinc (Zn^2+^) are crucial for T-cell signaling. Upon activation, T-cells undergo rapid calcium influx, which triggers the activation of calcineurin, a phosphatase that activates the NFAT (Nuclear Factor of Activated T-cells) transcription factor. NFAT then translocates to the nucleus and promotes the expression of cytokines such as IL-2, which are essential for T-cell proliferation and survival (90, 91). Nanomaterials that release calcium ions can enhance this signaling cascade, leading to more robust T-cell activation. Calcium-releasing nanoparticles, such as calcium phosphate nanocarriers, have been shown to enhance T-cell receptor (TCR) signaling by modulating intracellular calcium levels. This can lead to increased cytokine production and enhanced proliferation of CD8+ cytotoxic T-cells, which are essential for killing tumor cells. In bone cancer models, these nanoparticles have been used in combination with immune checkpoint inhibitors to improve T-cell responses and overcome the immune-suppressive environment of the bone TME.

Magnesium and zinc ions are crucial for T-cell proliferation. Magnesium is indispensable for maintaining the stability of TCRs on T-cells and participating in ATP metabolism, processes that support the energy requirements for T-cell proliferation. Studies have shown that in research on bone cancer, magnesium-doped nanoparticles enhance the proliferative capacity of T-cells within the TME, thereby improving the effectiveness of adoptive T-cell therapy. Zinc ions also play a vital role in T-cell function by modulating the activity of zinc-finger transcription factors involved in T-cell proliferation and survival. Zinc oxide nanoparticles have been utilized to increase intracellular zinc levels, which improves T-cell survival and function in the TME. Furthermore, these nanoparticles have demonstrated an ability to promote T-cell expansion and prevent exhaustion in preclinical models of CAR-T cell therapy for bone cancer.

Zinc-doped nanoparticles can reduce the expression of PD-1 on depleted T cells, which helps restore their function and improve their anti-cancer ability. In a bone cancer model, these nanoparticles combined with anti-PD-1 therapy showed enhanced T cell function and better tumor regression. By reducing immune checkpoint molecules and counteracting T-cell depletion, metal ion-based nanomaterials could significantly improve the effectiveness of immunotherapies.

A major challenge in bone cancer is the limitation of T cell infiltration into the tumor. Nanomaterials based on metal ions could help overcome this problem by modifying the tumor microenvironment (TME) to support T cell entry. For example, iron oxide nanoparticles can influence the production of chemokines such as CXCL9 and CXCL10, which attract T cells to tumors (92). These chemokines enhance the infiltration of CD8+ cytotoxic T cells into the TME, thereby improving immunotherapy outcomes. In addition, the nanoparticles release calcium.

## 6 Clinical applications and future prospects

Preclinical research has shown significant promise in using metal ions and nanomaterials for bone cancer treatment (93, 94). Studies have explored how these materials can modulate the immune system, enhance drug delivery, and target tumor cells directly (95). For instance, gold nanoparticles (AuNPs) have been used in preclinical models of osteosarcoma to deliver chemotherapeutic agents and immune checkpoint inhibitors, demonstrating enhanced tumor regression compared to traditional treatments (96). Gold nanoparticles’ photothermal properties enable targeted tumor cell destruction through near-infrared light, inducing localized heating and immune activation via tumor antigen release (97). In a similar fashion, researchers have examined zinc-doped nanoparticles for their potential to influence the immune microenvironment associated with bone cancer (98). Zinc influences macrophage polarization, promoting a transition from an immune-suppressive M2 phenotype to a pro-inflammatory M1 phenotype, which is crucial for an effective anti-tumor immune response (99). In preclinical osteosarcoma models, zinc-based nanoparticles have improved the efficacy of immune checkpoint blockade therapies by boosting T-cell activation and macrophage-mediated phagocytosis of tumor cells.

Despite these promising results, transitioning from preclinical research to clinical trials is still in its early stages. Nanoparticles made of iron oxide, widely utilized as contrast agents in MRI, are currently being investigated for their promise as theranostic agents that integrate both diagnostic and therapeutic capabilities. In bone cancer, iron oxide nanoparticles can be engineered to deliver drugs or immune modulators while enhancing imaging contrast for real-time treatment monitoring (100). Early-phase clinical trials have begun exploring metal-based nanoparticles in solid tumors, though specific trials for bone cancers remain limited. For instance, gold nanoparticles combined with radiation therapy are being tested in other cancer types, with potential adaptation for localized bone metastases (101).

The future of metal ion and nanomaterial-based therapies lies in their application in personalized and precision medicine. Bone cancer, like other cancers, exhibits significant heterogeneity in immune profiles, tumor microenvironments, and genetic mutations. Tailoring metal ion-nanomaterial systems to individual patients based on these characteristics could lead to more effective, targeted treatments (102). Assessing the immune profile of a patient, which encompasses T-cells, macrophages, and dendritic cells found in the tumor, might allow for the development of therapies based on metal ions that more efficiently regulate the immune system. For instance, patients with high levels of immune-suppressive tumor-associated macrophages (TAMs) could benefit from zinc-based nanoparticles, reprogramming macrophages to a pro-inflammatory M1 phenotype. Patients with T-cell exhaustion might benefit from magnesium-doped nanoparticles, which enhance T-cell activation and proliferation (103). Functionalizing nanomaterials with ligands targeting specific receptors overexpressed on bone cancer cells, such as integrins or VEGF receptors, could improve treatment specificity (104). For example, RGD-peptide functionalized nanoparticles could target integrins in osteosarcoma, delivering metal ions and drugs directly to the tumor while sparing healthy tissue.

Advances in genomic technologies enable the identification of specific mutations and alterations in bone cancer cells that could be targeted by metal ion-nanomaterial therapies (105). For example, mutations in the PI3K/AKT signaling pathway, crucial for cancer cell survival, could be targeted using nanoparticles designed to disrupt these pathways. Biomarker-driven treatments using metal ion-nanomaterial systems hold promise for increasing bone cancer immunotherapy efficacy (106). The combination of immune profiling, targeting specific tumors, and analyzing genomic data may result in personalized treatment strategies that enhance therapeutic results and reduce adverse effects.

Challenges and limitations must be addressed before metal ion and nanomaterial-based therapies can become widely available for bone cancer patients. Concerns include toxicity, biocompatibility, and the long-term effects of metal ions and nanoparticles in the body (107). Toxicity is a primary concern, as excessive metal ion accumulation can lead to oxidative stress and cell death. For example, zinc overload can disrupt cellular homeostasis, and silver nanoparticles can induce cytotoxicity in healthy cells at high concentrations (108). Controlled release of metal ions only in the tumor vicinity is critical for minimizing off-target effects. Biocompatibility is also a concern, particularly with metallic nanoparticles that may remain in the body for extended periods, potentially leading to organ damage or immune reactions (109). Research is focused on developing biodegradable nanomaterials that can be safely broken down and eliminated from the body.

The long-term effects of metal ions and nanoparticles remain unclear, as most studies have focused on short-term efficacy and toxicity. Long-term research is essential to evaluate nanoparticle persistence in tissues, potential chronic inflammation, and the risk of secondary cancers from DNA damage caused by metal ion-induced oxidative stress (110). Addressing drug resistance also poses a significant challenge in bone cancer treatment. Metal ion-based immunotherapies present potential solutions by targeting the immune system rather than cancer cells directly, such as enhancing T-cell activity or reprogramming TAMs to overcome immune evasion mechanisms employed by tumors (111). Ensuring sustained effectiveness will require ongoing investigation into adaptive resistance mechanisms.

## 7 Conclusion and discussion

The integration of metal ions and nanomaterials into cancer immunotherapy represents a promising frontier, especially for aggressive and challenging cancers like bone cancer. Conventional therapies, including surgical procedures, chemotherapy, and radiation therapy, frequently do not adequately tackle the intricacies of bone cancer, which involve elevated recurrence rates, the spread of cancer, and immunosuppressive TMEs. Immunotherapies that harness the body’s immune system to fight cancer have shown promise but face limitations in bone cancer due to their unique biological and structural challenges.

Metal ions and nanomaterials offer unique advantages, including targeted drug delivery, immune system modulation, and tumor microenvironment remodeling. Essential for immune cell signaling, T cell activation, and macrophage polarization, metal ions like calcium, magnesium, and zinc play a critical role in facilitating effective anti-tumor responses. Nanomaterials have the capability to accurately transport these metal ions to the tumor location and manage their release according to particular environmental triggers, including variations in pH or oxidative stress within the tumor microenvironment. The integration of metal ions and nanomaterials holds promise in improving the specificity and effectiveness of immunotherapy for bone cancer. By targeting the tumor immune microenvironment (TME), these strategies can enhance immune responses while minimizing off-target effects. Targeting immune mechanisms, such as immune checkpoint modulation and immune cell activation, is crucial for the success of cancer immunotherapies. In the context of bone cancer, the TME is often characterized by immune resistance and immune cell dysfunction, which hinder the efficacy of traditional treatments. Nanomaterials and metal ions have the potential to reprogram the TME, promoting anti-tumor immune responses and overcoming resistance mechanisms.

In recent years, immunotherapy has shown unique clinical translational potential in the field of sarcoma, but its efficacy is limited by the highly heterogeneous nature of TME (112). The collaborative strategy of metal ions and nanomaterials proposed in this paper is highly consistent with the idea of transforming “cold tumor” into “hot tumor” in the immunotherapy of sarcoma. For example, the presence of B-cell infiltration and tertiary lymphoid structure (TLS) was found in sarcoma studies to predict immune checkpoint inhibitor response, and the zn-doped nanoparticles in this study may mimic similar effects by modulating macrophage polarization (M1/M2 balance) and enhancing dendritic cell antigen presentation. In addition, sarcoma clinical trials (such as SARCO28) have shown that PD-L1 expression is correlated with tumor-infiltrating lymphocyte (TIL) density, but with tumor-specific differences. In this study, PH-responsive nanocarriers can target the release of calcium ions to enhance T cell activation and may overcome similar spatial immunosuppression barriers in bone cancer. Future studies need to be combined with sarcoma immune classification systems to further explore the potential of metal ion-nanomaterial platforms in personalized therapy.

The combination of metal ions and nanomaterials has led to major breakthroughs. For example, gold nanoparticles can provide immune checkpoint inhibitors or chemotherapy drugs, while also providing photothermal properties to destroy cancer cells. When combined with metal ions such as calcium or zinc, these nanoparticles can modulate the activity of immune cells, enhancing the activity of T cells and dendritic cells in the TME. This synergistic effect both directly destroys cancer cells and strengthens the immune system.

Next-generation nanomaterials must be more efficient, safer, and more versatile. Multifunctional nanoparticles that carry multiple therapeutics in a single delivery system can deliver drugs and immunomodulators simultaneously. The safe decomposition of biodegradable nanomaterials in the tumor microenvironment, stimulating the release of therapeutic payloads from reactive nanoparticles, is also a promising area of research.

Improving safety is critical, as the transport of metal ions must be controlled to avoid toxicity. Future research should focus on metal-doped nanomaterials with controlled ion release, and explore novel metal ions or combinations with lower toxicity. Combining immunotherapies with nanomaterial-based drug delivery offers potential, such as combining immune checkpoint inhibitors with metal ion-loaded nanoparticles, to enhance immune responses and improve T cell-based therapies.

Antimicrobial resistance remains a major challenge. Metal-ion nanomaterial systems can help overcome drug resistance by targeting the immune system. For instance, modifying TAM or improving T cell function through metal ion therapies could lead to a treatment response that is both more effective and longer-lasting.

Metal ions and nanomaterials hold great promise for the treatment of bone cancer. Future research should focus on designing new nanomaterials, assessing safety and efficacy, and developing combination therapies to advance this field. Addressing drug resistance and ensuring long-term effectiveness are critical for translating these innovations into clinical practice. Integrating metal ions and nanomaterials into cancer immunotherapy offers new hope for patients resistant to traditional treatments. The future of bone cancer therapy lies in the synergy between immunotherapy and nanotechnology, continuously exploring breakthroughs in cancer treatment.

In conclusion, the integration of metal ions and nanomaterials in immunotherapy represents a promising strategy for overcoming the limitations of traditional bone cancer treatments. Key applications of metal ions, such as calcium, magnesium, and zinc, in modulating the immune response and improving the tumor microenvironment have shown great potential in enhancing immune cell activation and tumor targeting. Similarly, nanomaterials hold promise in improving drug delivery systems, facilitating the controlled release of therapeutic agents, and enhancing the effectiveness of immune checkpoint inhibitors. Together, these strategies could revolutionize the treatment landscape for bone cancer, offering more targeted and personalized approaches.

## References

[1] Biermann JS Adkins D Benjamin R Brigman B Chow W Conrad EU 3rd. Bone cancer. J Natl Compr Canc Netw. (2007) 5:420–37. doi: 10.6004/jnccn.2007.0037 17442233

[2] Biermann JS Adkins DR Agulnik M Benjamin RS Brigman B Butrynski JE. Bone cancer. J Natl Compr Canc Netw. (2013) 11:688–723. doi: 10.6004/jnccn.2013.0088 23744868

[3] Wang K Gu Y Liao Y Bang S Donnelly CR Chen O. PD-1 blockade inhibits osteoclast formation and murine bone cancer pain. J Clin Invest. (2020) 130:3603–20. doi: 10.1172/JCI133334 PMC7324182 32484460

[4] Meltzer PS Helman LJ. New horizons in the treatment of osteosarcoma. N Engl J Med. (2021) 385:2066–76. doi: 10.1056/NEJMra2103423 34818481

[5] Landi L D’Incà F Gelibter A Chiari R Grossi F Delmonte A. Bone metastases and immunotherapy in patients with advanced non-small-cell lung cancer. J Immunother Cancer. (2019) 7:316. doi: 10.1186/s40425-019-0793-8 31752994 PMC6868703

[6] Harwood AR Krajbich JI Fornasier VL. Radiotherapy of chondrosarcoma of bone. Cancer. (1980) 45:2769–77. doi: 10.1002/1097-0142(19800601)45:11<2769::AID-CNCR2820451111>3.0.CO;2-X 7379008

[7] Randall RL Gowski W. Grade 1 chondrosarcoma of bone: a diagnostic and treatment dilemma. J Natl Compr Canc Netw. (2005) 3:149–56. doi: 10.6004/jnccn.2005.0011 19817027

[8] Ali A Hoyle A Haran ÁM Brawley CD Cook A Amos C. Association of bone metastatic burden with survival benefit from prostate radiotherapy in patients with newly diagnosed metastatic prostate cancer: A secondary analysis of a randomized clinical trial. JAMA Oncol. (2021) 7:555–63. doi: 10.1001/jamaoncol.2020.7857 PMC7893550 33599706

[9] Yin JJ Pollock CB Kelly K. Mechanisms of cancer metastasis to the bone. Cell Res. (2005) 15:57–62. doi: 10.1038/sj.cr.7290266 15686629

[10] Coleman RE Croucher PI Padhani AR Clézardin P Chow E Fallon M. Bone metastases. Nat Rev Dis Primers. (2020) 6:83. doi: 10.1038/s41572-020-00216-3 33060614

[11] Hofbauer LC Bozec A Rauner M Jakob F Perner S Pantel K. Novel approaches to target the microenvironment of bone metastasis. Nat Rev Clin Oncol. (2021) 18:488–505. doi: 10.1038/s41571-021-00499-9 33875860

[12] Ban J Fock V Aryee DNT Kovar H. Mechanisms, diagnosis and treatment of bone metastases. Cells. (2021) 10(11):2944. doi: 10.3390/cells10112944 34831167 PMC8616226

[13] Weiss MC Eulo V Van Tine BA. Truly man’s best friend: canine cancers drive drug repurposing in osteosarcoma. Clin Cancer Res. (2022) 28:571–2. doi: 10.1158/1078-0432.CCR-21-3471 34880110

[14] Yuan P Min Y Zhao Z. Multifunctional nanoparticles for the treatment and diagnosis of osteosarcoma. Biomater Adv. (2023) 151:213466. doi: 10.1016/j.bioadv.2023.213466 37229927

[15] LaMarche NM Hegde S Park MD Maier BB Troncoso L Le Berichel J. An IL-4 signalling axis in bone marrow drives pro-tumorigenic myelopoiesis. Nature. (2024) 625:166–74. doi: 10.1038/s41586-023-06797-9 PMC11189607 38057662

[16] Wang DR Wu XL Sun YL. Therapeutic targets and biomarkers of tumor immunotherapy: response versus non-response. Signal Transduct Target Ther. (2022) 7:331. doi: 10.1038/s41392-022-01136-2 36123348 PMC9485144

[17] Siozopoulou V Smits E Zwaenepoel K Liu J Pouliakis A Pauwels PA. PD-1, PD-L1, IDO, CD70 and microsatellite instability as potential targets to prevent immune evasion in sarcomas. Immunotherapy. (2023) 15:1257–73. doi: 10.2217/imt-2022-0049 37661910

[18] Kang J La Manna F Bonollo F Sampson N Alberts IL Mingels C. Tumor microenvironment mechanisms and bone metastatic disease progression of prostate cancer. Cancer Lett. (2022) 530:156–69. doi: 10.1016/j.canlet.2022.01.015 35051532

[19] Wu C Gong S Duan Y Deng C Kallendrusch S Berninghausen L. A tumor microenvironment-based prognostic index for osteosarcoma. J BioMed Sci. (2023) 30:23. doi: 10.1186/s12929-023-00917-3 37055822 PMC10099847

[20] Hu Y Feng J Gu T Wang L Wang Y Zhou L. CAR T-cell therapies in China: rapid evolution and a bright future. Lancet Haematol. (2022) 9:e930–41. doi: 10.1016/S2352-3026(22)00291-5 36455608

[21] Jain T Olson TS Locke FL. How I treat cytopenias after CAR T-cell therapy. Blood. (2023) 141:2460–9. doi: 10.1182/blood.2022017415 PMC10646792 36800563

[22] Flugel CL Majzner RG Krenciute G Dotti G Riddell SR Wagner DL. Overcoming on-target, off-tumour toxicity of CAR T cell therapy for solid tumours. Nat Rev Clin Oncol. (2023) 20:49–62. doi: 10.1038/s41571-022-00704-3 36418477 PMC10278599

[23] Li Z Zhang W Zhang Z Gao H Qin Y. Cancer bone metastases and nanotechnology-based treatment strategies. Expert Opin Drug Delivery. (2022) 19:1217–32. doi: 10.1080/17425247.2022.2093856 35737871

[24] Hao X Jiang B Wu J Xiang D Xiong Z Li C. Nanomaterials for bone metastasis. J Control Release. (2024) 373:640–51. doi: 10.1016/j.jconrel.2024.07.067 39084467

[25] Zhang P Meng J Li Y Yang C Hou Y Tang W. Nanotechnology-enhanced immunotherapy for metastatic cancer. Innovation (Camb). (2021) 2:100174. doi: 10.1016/j.xinn.2021.100174 34766099 PMC8571799

[26] Yang H Yu Z Ji S Huo Q Yan J Gao Y. Targeting bone microenvironments for treatment and early detection of cancer bone metastatic niches. J Control Release. (2022) 341:443–56. doi: 10.1016/j.jconrel.2021.11.005 34748870

[27] Li J Wang Q Han Y Jiang L Lu S Wang B. Development and application of nanomaterials, nanotechnology and nanomedicine for treating hematological Malignancies. J Hematol Oncol. (2023) 16:65. doi: 10.1186/s13045-023-01460-2 37353849 PMC10290401

[28] Chen LJ Kaji H. Modeling angiogenesis with micro- and nanotechnology. Lab Chip. (2017) 17:4186–219. doi: 10.1039/C7LC00774D 28981128

[29] Krzywoszyńska K Witkowska D Swiatek-Kozlowska J Szebesczyk A Kozłowski H. General aspects of metal ions as signaling agents in health and disease. Biomolecules. (2020) 10(10):1417. doi: 10.3390/biom10101417 33036384 PMC7600656

[30] Shen F Fang Y Wu Y Zhou M Shen J Fan X. Metal ions and nanometallic materials in antitumor immunity: Function, application, and perspective. J Nanobiotechnol. (2023) 21:20. doi: 10.1186/s12951-023-01771-z PMC9850565 36658649

[31] Stelling MP Motta JM Mashid M Johnson WE Pavão MS Farrell NP. Metal ions and the extracellular matrix in tumor migration. FEBS J. (2019) 286:2950–64. doi: 10.1111/febs.v286.15 31379111

[32] Chen B Yu P Chan WN Xie F Zhang Y Liang L. Cellular zinc metabolism and zinc signaling: from biological functions to diseases and therapeutic targets. Signal Transduct Target Ther. (2024) 9:6. doi: 10.1038/s41392-023-01679-y 38169461 PMC10761908

[33] Hübner C Haase H. Interactions of zinc- and redox-signaling pathways. Redox Biol. (2021) 41:2285. doi: 10.1016/j.redox.2021.101916 PMC7937829 33662875

[34] Maret W. Zinc in cellular regulation: the nature and significance of “Zinc signals”. Int J Mol Sci. (2017) 18(11):2285. doi: 10.3390/ijms18112285 29088067 PMC5713255

[35] Qin H Weng J Zhou B Zhang W Li G Chen Y. Magnesium ions promote *in vitro* rat bone marrow stromal cell angiogenesis through notch signaling. Biol Trace Elem Res. (2023) 201:2823–42. doi: 10.1007/s12011-022-03364-7 35870071

[36] Dai Y Wu J Wang J Wang H Guo B Jiang T. Magnesium ions promote the induction of immunosuppressive bone microenvironment and bone repair through HIF-1α-TGF-β Axis in dendritic cells. Small. (2024) 20:e2311344. doi: 10.1002/smll.202311344 38661278

[37] Jiang Y Ding P. Calcium signaling in plant immunity: a spatiotemporally controlled symphony. Trends Plant Sci. (2023) 28:74–89. doi: 10.1016/j.tplants.2022.11.001 36504136

[38] Concepcion AR Wagner LE 2nd Zhu J Tao AY Yang J Khodadadi-Jamayran A. The volume-regulated anion channel LRRC8C suppresses T cell function by regulating cyclic dinucleotide transport and STING-p53 signaling. Nat Immunol. (2022) 23:287–302. doi: 10.1038/s41590-021-01105-x 35105987 PMC8991407

[39] Dodd AN Kudla J Sanders D. The language of calcium signaling. Annu Rev Plant Biol. (2010) 61:593–620. doi: 10.1146/annurev-arplant-070109-104628 20192754

[40] Cheng HP Wei S Wei LP Verkhratsky A. Calcium signaling in physiology and pathophysiology. Acta Pharmacol Sin. (2006) 27:767–72. doi: 10.1111/j.1745-7254.2006.00399.x 16787558

[41] Zusso M Lunardi V Franceschini D Pagetta A Lo R Stifani S. Ciprofloxacin and levofloxacin attenuate microglia inflammatory response via TLR4/NF-kB pathway. J Neuroinflamm. (2019) 16:148. doi: 10.1186/s12974-019-1538-9 PMC6637517 31319868

[42] Godson DL Campos M Babiuk LA. Non-major histocompatibility complex-restricted cytotoxicity of bovine coronavirus-infected target cells mediated by bovine intestinal intraepithelial leukocytes. J Gen Virol. (1991) 72:2457–65. doi: 10.1099/0022-1317-72-10-2457 1655957

[43] Ahokas RA Warrington KJ Gerling IC Sun Y Wodi LA Herring PA. Aldosteronism and peripheral blood mononuclear cell activation: a neuroendocrine-immune interface. Circ Res. (2003) 93:e124–135. doi: 10.1161/01.RES.0000102404.81461.25 PMC2896314 14576195

[44] Voelkl J Tuffaha R Luong TTD Zickler D Masyout J Feger M. Zinc inhibits phosphate-induced vascular calcification through TNFAIP3-mediated suppression of NF-κB. J Am Soc Nephrol. (2018) 29:1636–48. doi: 10.1681/ASN.2017050492 PMC6054342 29654213

[45] Zheng J Chen T Wang K Peng C Zhao M Xie Q. Engineered multifunctional zinc-organic framework-based aggregation-induced emission nanozyme for accelerating spinal cord injury recovery. ACS Nano. (2024) 18:2355–69. doi: 10.1021/acsnano.3c10541 38197586

[46] Lupu-Meiri M Silver RB Simons AH Gershengorn MC Oron Y. Constitutive signaling by Kaposi’s sarcoma-associated herpesvirus G-protein-coupled receptor desensitizes calcium mobilization by other receptors. J Biol Chem. (2001) 276:7122–8. doi: 10.1074/jbc.M006359200 11116138

[47] Mueller A Mahmoud NG Goedecke MC McKeating JA Strange PG. Pharmacological characterization of the chemokine receptor, CCR5. Br J Pharmacol. (2002) 135:1033–43. doi: 10.1038/sj.bjp.0704540 PMC1573204 11861332

[48] Sun Y Sukumaran P Singh BB. Magnesium-induced cell survival is dependent on TRPM7 expression and function. Mol Neurobiol. (2020) 57:528–38. doi: 10.1007/s12035-019-01713-7 PMC6968994 31392516

[49] Zocchi M Locatelli L Zuccotti GV Mazur A Béchet D Maier JA. Magnesium homeostasis in myogenic differentiation-A focus on the regulation of TRPM7, magT1 and SLC41A1 transporters. Int J Mol Sci. (2022) 23(3):1658. doi: 10.3390/ijms23031658 35163580 PMC8836031

[50] Merino-Wong M Niemeyer BA Alansary D. Plasma membrane calcium ATPase regulates stoichiometry of CD4(+) T-cell compartments. Front Immunol. (2021) 12:687242. doi: 10.3389/fimmu.2021.687242 34093590 PMC8175910

[51] Erhardt B Marcora MS Frenkel L Bochicchio PA Bodin DH Silva BA. Plasma membrane calcium ATPase downregulation in dopaminergic neurons alters cellular physiology and motor behaviour in Drosophila melanogaster. Eur J Neurosci. (2021) 54:5915–31. doi: 10.1111/ejn.15401 34312939

[52] Wahab S Salman A Khan Z Khan S Krishnaraj C Yun SI. Metallic nanoparticles: A promising arsenal against antimicrobial resistance-unraveling mechanisms and enhancing medication efficacy. Int J Mol Sci. (2023) 24(19):14897. doi: 10.3390/ijms241914897 37834344 PMC10573543

[53] Alphandéry E. Natural metallic nanoparticles for application in nano-oncology. Int J Mol Sci. (2020) 21(12):4412. doi: 10.3390/ijms21124412 32575884 PMC7352233

[54] Kang Y Xu C Meng L Dong X Qi M Jiang D. Exosome-functionalized magnesium-organic framework-based scaffolds with osteogenic, angiogenic and anti-inflammatory properties for accelerated bone regeneration. Bioact Mater. (2022) 18:26–41. doi: 10.1016/j.bioactmat.2022.02.012 35387167 PMC8961306

[55] Patel A Zaky SH Schoedel K Li H Sant V Beniash E. Design and evaluation of collagen-inspired mineral-hydrogel nanocomposites for bone regeneration. Acta Biomater. (2020) 112:262–73. doi: 10.1016/j.actbio.2020.05.034 PMC7446305 32497742

[56] Lin Z Liu Y Ma X Hu S Zhang J Wu Q. Photothermal ablation of bone metastasis of breast cancer using PEGylated multi-walled carbon nanotubes. Sci Rep. (2015) 5:11709. doi: 10.1038/srep11709 26122018 PMC4485034

[57] Luo F Li R Zheng H Xu Y Yang L Qu C. Differentiation of bone mesenchymal stem cells into vascular endothelial cell-like cells using functionalized single-walled carbon nanotubes. Front Bioeng Biotechnol. (2022) 10:913080. doi: 10.3389/fbioe.2022.913080 35747494 PMC9209768

[58] Gao X Li L Cai X Huang Q Xiao J Cheng Y. Targeting nanoparticles for diagnosis and therapy of bone tumors: Opportunities and challenges. Biomaterials. (2021) 265:120404. doi: 10.1016/j.biomaterials.2020.120404 32987273

[59] Lee H Shields AF Siegel BA Miller KD Krop I Ma CX. (64)Cu-MM-302 positron emission tomography quantifies variability of enhanced permeability and retention of nanoparticles in relation to treatment response in patients with metastatic breast cancer. Clin Cancer Res. (2017) 23:4190–202. doi: 10.1158/1078-0432.CCR-16-3193 PMC6790129 28298546

[60] Dozono H Yanazume S Nakamura H Etrych T Chytil P Ulbrich K. HPMA copolymer-conjugated pirarubicin in multimodal treatment of a patient with stage IV prostate cancer and extensive lung and bone metastases. Target Oncol. (2016) 11:101–6. doi: 10.1007/s11523-015-0379-4 26194363

[61] Mateu-Sanz M Ginebra MP Tornín J Canal C. Cold atmospheric plasma enhances doxorubicin selectivity in metastasic bone cancer. Free Radic Biol Med. (2022) 189:32–41. doi: 10.1016/j.freeradbiomed.2022.07.007 35843475

[62] Judson I Radford JA Harris M Blay JY van Hoesel Q le Cesne A. Randomised phase II trial of pegylated liposomal doxorubicin (DOXIL/CAELYX) versus doxorubicin in the treatment of advanced or metastatic soft tissue sarcoma: a study by the EORTC Soft Tissue and Bone Sarcoma Group. Eur J Cancer. (2001) 37:870–7. doi: 10.1016/S0959-8049(01)00050-8 11313175

[63] Zhao L Wen X Xu W Pang Y Sun L Wu X. Clinical evaluation of (68)Ga-FAPI-RGD for imaging of fibroblast activation protein and integrin α(v)β(3) in various cancer types. J Nucl Med. (2023) 64:1210–7. doi: 10.2967/jnumed.122.265383 PMC10394316 37142301

[64] Hernandez DS Schunk HC Shankar KM Rosales AM Suggs LJ. Poly-d-lysine coated nanoparticles to identify pro-inflammatory macrophages. Nanoscale Adv. (2020) 2:3849–57. doi: 10.1039/D0NA00373E PMC9416964 36132778

[65] Lorenzo-Anota HY Zarate-Triviño DG Uribe-Echeverría JA Ávila-Ávila A Rangel-López JR Martínez-Torres AC. Chitosan-coated gold nanoparticles induce low cytotoxicity and low ROS production in primary leucocytes, independent of their proliferative status. Pharmaceutics. (2021) 13(7):942. doi: 10.3390/pharmaceutics13070942 34202522 PMC8309170

[66] Khalifehzadeh R Arami H. Biodegradable calcium phosphate nanoparticles for cancer therapy. Adv Colloid Interface Sci. (2020) 279:102157. doi: 10.1016/j.cis.2020.102157 32330734 PMC7261203

[67] Sapino S Chindamo G Chirio D Manzoli M Peira E Riganti C. Calcium phosphate-coated lipid nanoparticles as a potential tool in bone diseases therapy. Nanomater (Basel). (2021) 11(11):2983. doi: 10.3390/nano11112983 PMC8625061 34835747

[68] Chu W Huang Y Yang C Liao Y Zhang X Yan M. Calcium phosphate nanoparticles functionalized with alendronate-conjugated polyethylene glycol (PEG) for the treatment of bone metastasis. Int J Pharm. (2017) 516:352–63. doi: 10.1016/j.ijpharm.2016.11.051 27887884

[69] Liguori A De Vita A Rossi G Dolci LS Panzavolta S Gualandi C. A modular composite device of poly(Ethylene oxide)/poly(Butylene terephthalate) (PEOT/PBT) nanofibers and gelatin as a dual drug delivery system for local therapy of soft tissue tumors. Int J Mol Sci. (2022) 23(6):3239. doi: 10.3390/ijms23063239 35328661 PMC8948985

[70] Yu F Wei J Cui X Yu C Ni W Bungert J. Post-translational modification of RNA m6A demethylase ALKBH5 regulates ROS-induced DNA damage response. Nucleic Acids Res. (2021) 49:5779–97. doi: 10.1093/nar/gkab415 PMC8191756 34048572

[71] Ingle J Uttam B Panigrahi R Khatua S Basu S. Dog-bone shaped gold nanoparticle-mediated chemo-photothermal therapy impairs the powerhouse to trigger apoptosis in cancer cells. J Mater Chem B. (2023) 11:9732–41. doi: 10.1039/D3TB01716H 37791575

[72] Sun P Qu F Zhang C Cheng P Li X Shen Q. NIR-II excitation phototheranostic platform for synergistic photothermal therapy/chemotherapy/chemodynamic therapy of breast cancer bone metastases. Adv Sci (Weinh). (2022) 9:e2204718. doi: 10.1002/advs.202204718 36216756 PMC9685450

[73] Sun W Ge K Jin Y Han Y Zhang H Zhou G. Bone-targeted nanoplatform combining zoledronate and photothermal therapy to treat breast cancer bone metastasis. ACS Nano. (2019) 13:7556–67. doi: 10.1021/acsnano.9b00097 31259530

[74] Zeng Y Pan Z Yuan J Song Y Feng Z Chen Z. Inhibiting osteolytic breast cancer bone metastasis by bone-targeted nanoagent via remodeling the bone tumor microenvironment combined with NIR-II photothermal therapy. Small. (2023) 19:e2301003. doi: 10.1002/smll.202301003 37211708

[75] Li H Li Y Jiao J Hu HM. Alpha-alumina nanoparticles induce efficient autophagy-dependent cross-presentation and potent antitumour response. Nat Nanotechnol. (2011) 6:645–50. doi: 10.1038/nnano.2011.153 PMC3483867 21926980

[76] Li X Hufnagel S Xu H Valdes SA Thakkar SG Cui Z. Aluminum (Oxy)Hydroxide nanosticks synthesized in bicontinuous reverse microemulsion have potent vaccine adjuvant activity. ACS Appl Mater Interfaces. (2017) 9:22893–901. doi: 10.1021/acsami.7b03965 PMC5556926 28621928

[77] Bansal V Kumar M Bhardwaj A Brahmne HG Singh H. *In vivo* efficacy and toxicity evaluation of polycaprolactone nanoparticles and aluminum based admixture formulation as vaccine delivery system. Vaccine. (2015) 33:5623–32. doi: 10.1016/j.vaccine.2015.08.076 26343498

[78] Dong H Wen ZF Chen L Zhou N Liu H Dong S. Polyethyleneimine modification of aluminum hydroxide nanoparticle enhances antigen transportation and cross-presentation of dendritic cells. Int J Nanomed. (2018) 13:3353–65. doi: 10.2147/IJN.S164097 PMC5995426 29922056

[79] Zhao H Xu J Li Y Guan X Han X Xu Y. Nanoscale coordination polymer based nanovaccine for tumor immunotherapy. ACS Nano. (2019) 13:13127–35. doi: 10.1021/acsnano.9b05974 31710460

[80] Ahn S Lee IH Kang S Kim D Choi M Saw PE. Gold nanoparticles displaying tumor-associated self-antigens as a potential vaccine for cancer immunotherapy. Adv Healthc Mater. (2014) 3:1194–9. doi: 10.1002/adhm.201300597 24652754

[81] Afroz S Medhi H Maity S Minhas G Battu S Giddaluru J. Mesoporous ZnO nanocapsules for the induction of enhanced antigen-specific immunological responses. Nanoscale. (2017) 9:14641–53. doi: 10.1039/C7NR03697C 28936523

[82] Ridolfi R Riccobon A Galassi R Giorgetti G Petrini M Fiammenghi L. Evaluation of *in vivo* labelled dendritic cell migration in cancer patients. J Transl Med. (2004) 2:27. doi: 10.1186/1479-5876-2-27 15285807 PMC509425

[83] Lv X Huang J Min J Wang H Xu Y Zhang Z. Multi-signaling pathway activation by pH responsive manganese particles for enhanced vaccination. J Control Release. (2023) 357:109–19. doi: 10.1016/j.jconrel.2023.01.078 36738971

[84] Zheng SJ Yang M Luo JQ Liu R Song J Chen Y. Manganese-based immunostimulatory metal-organic framework activates the cGAS-STING pathway for cancer metalloimmunotherapy. ACS Nano. (2023) 17:15905–17. doi: 10.1021/acsnano.3c03962 37565626

[85] Lei J Zhang W Ma L He Y Liang H Zhang X. Sonodynamic amplification of cGAS-STING activation by cobalt-based nanoagonist against bone and metastatic tumor. Biomaterials. (2023) 302:122295. doi: 10.1016/j.biomaterials.2023.122295 37666101

[86] Chen H Zhang Y Li L Guo R Shi X Cao X. Effective cpG delivery using zwitterion-functionalized dendrimer-entrapped gold nanoparticles to promote T cell-mediated immunotherapy of cancer cells. Biosensors (Basel). (2022) 12(2):71. doi: 10.3390/bios12020071 35200332 PMC8869692

[87] He N Jiang J. Contribution of immune cells to bone metastasis pathogenesis. Front Endocrinol (Lausanne). (2022) 13:1019864. doi: 10.3389/fendo.2022.1019864 36246916 PMC9556850

[88] Schirrmacher V. Cancer-reactive memory T cells from bone marrow: Spontaneous induction and therapeutic potential (Review). Int J Oncol. (2015) 47:2005–16. doi: 10.3892/ijo.2015.3197 26459860

[89] Huo S Liu S Liu Q Xie E Miao L Meng X. Copper-zinc-doped bilayer bioactive glasses loaded hydrogel with spatiotemporal immunomodulation supports MRSA-infected wound healing. Adv Sci (Weinh). (2024) 11:e2302674. doi: 10.1002/advs.202302674 38037309 PMC10837387

[90] Deng Z Loyher PL Lazarov T Li L Shen Z Bhinder B. The nuclear factor ID3 endows macrophages with a potent anti-tumour activity. Nature. (2024) 626:864–73. doi: 10.1038/s41586-023-06950-4 PMC10881399 38326607

[91] Michaels AJ Campbell C Bou-Puerto R Rudensky AY. Nuclear receptor LXRβ controls fitness and functionality of activated T cells. J Exp Med. (2021) 218(4). doi: 10.1084/jem.20201311 PMC7774588 33373442

[92] Pusic K Aguilar Z McLoughlin J Kobuch S Xu H Tsang M. Iron oxide nanoparticles as a clinically acceptable delivery platform for a recombinant blood-stage human malaria vaccine. Faseb J. (2013) 27(3):1153–66. doi: 10.1096/fj.12-218362 PMC3574285 23195035

[93] Egodawatte S Greenstein KE Vance I Rivera E Myung NV Parkin GF. Electrospun hematite nanofiber/mesoporous silica core/shell nanomaterials as an efficient adsorbent for heavy metals. Rsc Adv. (2016) 6:90516–25. doi: 10.1039/C6RA19876G

[94] Jayaraman Shrisudersan Tang Wei Yongsunthon Ruchirej. Electrochemical synthesis of M:DNA nanohybrids. J Electrochemical Soc. (2011) 158(5):K123. doi: 10.1149/1.3559474

[95] Eman A Lukasz W Annj Z Bernadine I Lucy DS. Functionalized scaffolds for tissue engineering. Front Bioengineering Biotechnol. (2016) 4. doi: 10.3389/conf.FBIOE.2016.01.02145

[96] Bai X Zhang J Chang YN Gu W Lei R Qin Y. Nanoparticles with high-surface negative-charge density disturb the metabolism of low-density lipoprotein in cells. Int J Mol ences. (2018) 19(9):2790. doi: 10.3390/ijms19092790 PMC6164102 30227604

[97] White SB Kim DH Guo Y Li W Yang Y Chen J. Biofunctionalized hybrid magnetic gold nanoparticles as catalysts for photothermal ablation of colorectal liver metastases. Radiology. (2017) 285(3):809–19. doi: 10.1148/radiol.2017161497 PMC5708290 28707960

[98] Au A Ms B Fa C Ls D Aa C Nas E. Synergistic effect of silver doped ZnO nanomaterials enhances the anticancer potential against A459 lung cancer cells - ScienceDirect. J King Saud Univ Sci. (2021) 34(1):101724. doi: 10.1016/j.jksus.2021.101724

[99] Zhu WQ Shao SY Xu LN Chen WQ Yu XY Tang KM. Enhanced corrosion resistance of zinc-containing nanowires-modified titanium surface under exposure to oxidizing microenvironment. J Nanobiotechnol. (2019) 17(1):55. doi: 10.1186/s12951-019-0488-9 PMC6466780 30992009

[100] Al-Jameel SS Almessiere MA Khan FA Taskhandi N Slimani Y Al-Saleh NS. Synthesis, characterization, anti-cancer analysis of sr(0.5)Ba(0.5)Dy(x)Sm(x)Fe(8-2x)O(19) (0.00 ≤ x ≤ 1.0) microsphere nanocomposites. Nanomater (Basel). (2021) 11(3):700. doi: 10.3390/nano11030700 PMC7998806 33799552

[101] Yang C Neshatian M Van Prooijen M Chithrani DB. Cancer nanotechnology: enhanced therapeutic response using peptide-modified gold nanoparticles. J Nanosci Nanotechnol. (2014) 14:4813–9. doi: 10.1166/jnn.2014.9280 24757948

[102] Ahmad FS French B Bowles KH Sevilla-Cazes J Jaskowiak-Barr A Gallagher TR. Incorporating patient-centered factors into heart failure readmission risk prediction: A mixed-methods study. Am Heart J. (2018) 200:75–82. doi: 10.1016/j.ahj.2018.03.002 29898852 PMC6004826

[103] Wang LJ Ni XH Zhang F Peng Z Yu FX Zhang LB. Osteoblast response to copper-doped microporous coatings on titanium for improved bone integration. Nanoscale Res Lett. (2021) 16:146. doi: 10.1186/s11671-021-03602-2 34542720 PMC8452820

[104] Liu Q Jin C Wang Y Fang X Zhang X Chen Z. Aptamer-conjugated nanomaterials for specific cancer cell recognition and targeted cancer therapy. NPG Asia Mater. (2014) 6:e95. doi: 10.1038/am.2014.12 29619132 PMC5880215

[105] Psioda MA Xu J Jiang Q Ke C Yang Z Ibrahim JG. Bayesian adaptive basket trial design using model averaging. Biostatistics. (2021) 22:19–34. doi: 10.1093/biostatistics/kxz014 31107534 PMC7846150

[106] Lin ZT Li Y Gu J Wang H Zhu Z Hong X. A conductive nanowire-mesh biosensor for ultrasensitive detection of serum C-reactive protein in melanoma. Advanced Funct Materials. (2018) 28(31):1802482.1-.9. doi: 10.1002/adfm.201802482

[107] Zhou H Tang D Kang X Yuan H Yu Y Xiong X. Degradable pseudo conjugated polymer nanoparticles with NIR-II photothermal effect and cationic quaternary phosphonium structural bacteriostasis for anti-infection therapy. Adv Sci (Weinh). (2022) 9:e2200732. doi: 10.1002/advs.202200732 35343113 PMC9165483

[108] Fizesan I Iacovita C Pop A Kiss B Dudric R Stiufiuc R. The effect of zn-substitution on the morphological, magnetic, cytotoxic, and *in vitro* hyperthermia properties of polyhedral ferrite magnetic nanoparticles. Pharmaceutics. (2021) 13(12):2148. doi: 10.3390/pharmaceutics13122148 34959431 PMC8708233

[109] Auciello O Renou S Kang K Tasat D Olmedo D. A biocompatible ultrananocrystalline diamond (UNCD) coating for a new generation of dental implants. Nanomater (Basel). (2022) 12(5):782. doi: 10.3390/nano12050782 PMC8911871 35269268

[110] Alarifi S Ali D Alkahtani S. Nanoalumina induces apoptosis by impairing antioxidant enzyme systems in human hepatocarcinoma cells. Int J Nanomed. (2015) 10:3751–60. doi: 10.2147/ijn.S82050 PMC4448921 26045665

[111] Liu JB Chen D Bao TT Fan FT Yu C. The anticancer effects of atractylenolide III associate with the downregulation of jak3/stat3-dependent IDO expression. Front Pharmacol. (2019) 10:1505. doi: 10.3389/fphar.2019.01505 32038231 PMC6983677

[112] Recine F Vanni S Bongiovanni A Fausti V Mercatali L Miserocchi G. Clinical and translational implications of immunotherapy in sarcomas. Front Immunol. (2024) 15:1378398. doi: 10.3389/fimmu.2024.1378398 38983859 PMC11231074

